# Case Report and Literature Review: Lumbar Disc Extrusion Misdiagnosed as an Epidural Hematoma

**DOI:** 10.7759/cureus.43115

**Published:** 2023-08-08

**Authors:** Fadi Nader, Georges F Bassil, Mohamad Ali Sleiman, Nicolas Nicolas

**Affiliations:** 1 Orthopedics and Trauma, Université Paris Cité, Paris, FRA; 2 Orthopedics and Traumatology, Lebanese University Faculty of Medicine, Beirut, LBN; 3 Orthopedic Surgery, Grand Hôpital de l'Est Francilien, Meaux, FRA

**Keywords:** cauda equina, signal intensity, mri, lumbar disc herniation, spinal epidural hematoma

## Abstract

Accurate differentiation between epidural hematomas and lumbar disc extrusion is essential due to the potential overlap in clinical presentations. We present a case report highlighting a significant challenge in which a massive lumbar disc extrusion was mistaken for an epidural hematoma.

This is a case report of a 38-year-old male patient who developed cauda equina syndrome four days after experiencing an audible cracking in the lower back during weightlifting activity. Magnetic resonance imaging (MRI) was inconclusive, unable to distinguish between an extruded nucleus pulposus and a spinal epidural hematoma. Subsequently, an urgent operation revealed a large herniated disc at the L4-L5 level, ruling out any hematoma. The patient's post-operative follow-up showed significant improvement, with almost complete recovery of motor and sensory functions.

This case emphasizes the challenges faced when distinguishing between epidural hematomas and lumbar disc herniations, particularly on MRI. The lumbar disc herniation's substantial size, cranial and caudal migration on multiple levels, and signal intensity contributed to the misdiagnosis, underscoring the importance of careful interpretation and awareness of such complexities.

## Introduction

The intervertebral discs consist of three main components: the inner nucleus pulposus (NP), the outer annulus fibrosus (AF), and the cartilaginous endplates that connect the disc to the adjacent vertebrae [1]. Disc herniation occurs when part or all of the nucleus pulposus protrudes through the annulus fibrosus. This can be due mainly to degenerative processes in the aging population. Among the other causes, such as connective tissue disorders and congenital malformations (e.g., short pedicles), traumatic disc herniation should not be missed [2]. The latter is a rare entity, especially in lumbar spinal trauma, due to the more vulnerable cancellous bone adjacent to the nucleus space, which usually leads to fractures instead of disc herniations in traumatic cases especially those caused by excessive axial load [3].

On the other hand, spinal epidural hematoma (SEH) could be another rare cause of acute neurologic symptoms after trauma, coagulation disorders, and surgical/invasive spinal acts. Its pathophysiology is often unclear, but in the lumbar spine, it may result from the rupture of the Batson vertebral venous plexus [4].

To make the diagnosis, magnetic resonance imaging (MRI) is the preferred modality with 75% sensitivity and 77% of specificity in detecting lumbar disc herniation [5]. Signal intensity (SI) varies based on disc water content. Traumatic disc herniations give high SI on T1-weighted and low SI on T2-weighted MRI. Spinal epidural hematomas give low SI on T1-weighted and high SI on T2-weighted MRI [6].

As a result, lumbar disk herniations that resemble the presentation of spinal epidural hematoma might go unnoticed and be inadequately documented.

We present here a case of traumatic disc extrusion mistaken as a hematoma on MRI in a 38-year-old male patient who was operated on the same day of the presentation with a good outcome at the follow-up.

## Case presentation

This is a case of a 38-year-old patient who presented to the emergency department (ER) complaining of moderate lower back pain radiating to the left leg (L5 and S1 dermatomes), saddle hypoesthesia (S2-S3-S4-S5), anorectal dyschezia, and sexual impotence, four days after lifting 160 kg during a deadlift activity.

At the initial accident, he experienced an audible cracking in the lower back, followed by dizziness, discomfort, and severe back pain radiating to the left leg that lasted only a few minutes. After the trauma, the patient presented to the ER, where X-rays and physical exams were normal, without any sign of radiculopathy, and he was discharged only on painkillers. Afterward, he developed neurologic symptoms that were progressive and led him again to the ED after four days.

On the second physical examination, the patient had weakness of left toes extension graded 3/5 on the Medical Research Council's (MRC) muscle power scale, left plantarflexion graded 2/5, ankle dorsiflexion was also weak and graded 3/5 and ankle plantarflexion was 2/5. Whereas knee and hip joints had a full range of motion and normal muscle strength. There was no incontinence, but the anal sphincter tone was weak, the bulbocavernous reflex was negative and the plantar reflex was normal. Sensory hypoesthesia was observed at the L5-S1 level in the left foot, and the patient was unable to perform a left heel rise, indicating S1 motor involvement.

**Figure 1:**
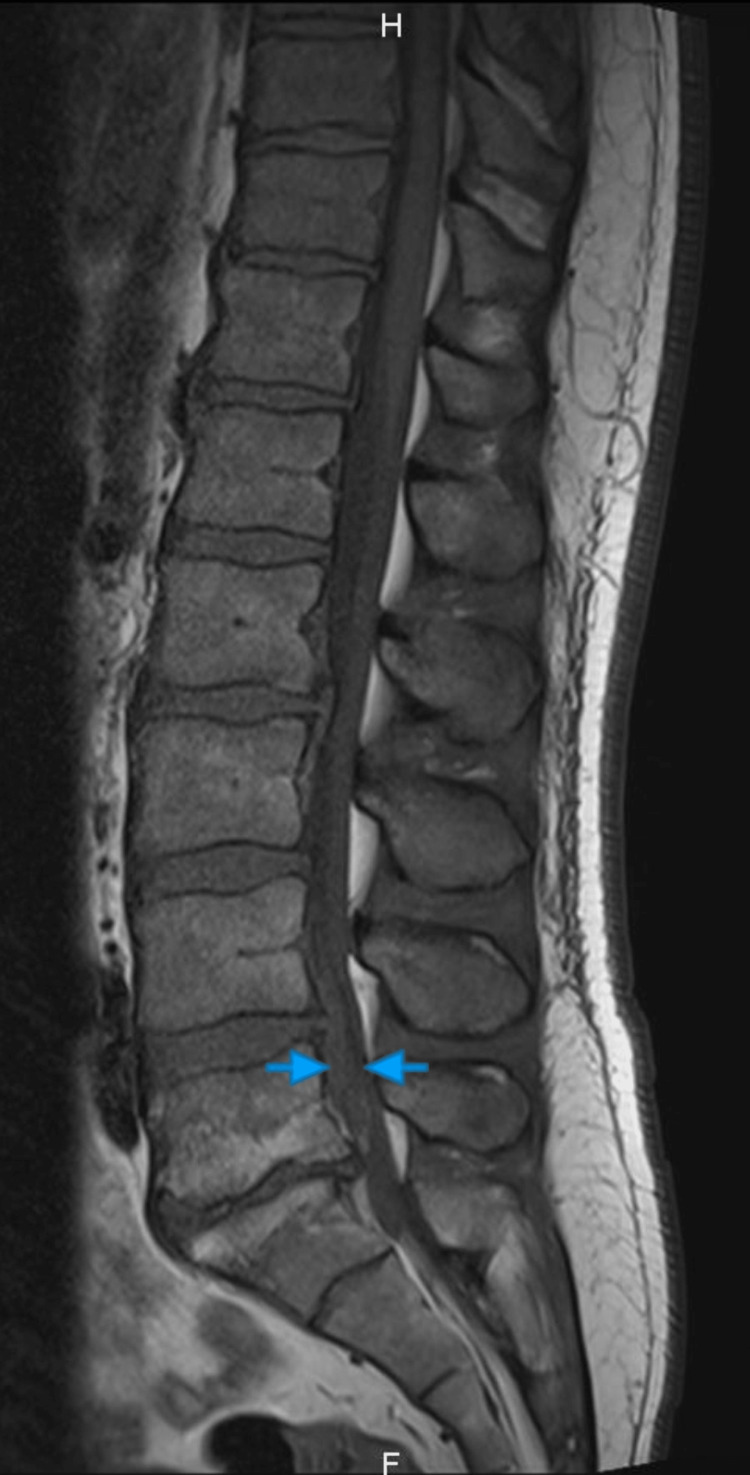
Sagittal T1-weighted image. Sagittal T1-weighted image showing spinal cord compression by an iso-intense structure at L4-L5 level, marked by two blue arrows on each side.

**Figure 2:**
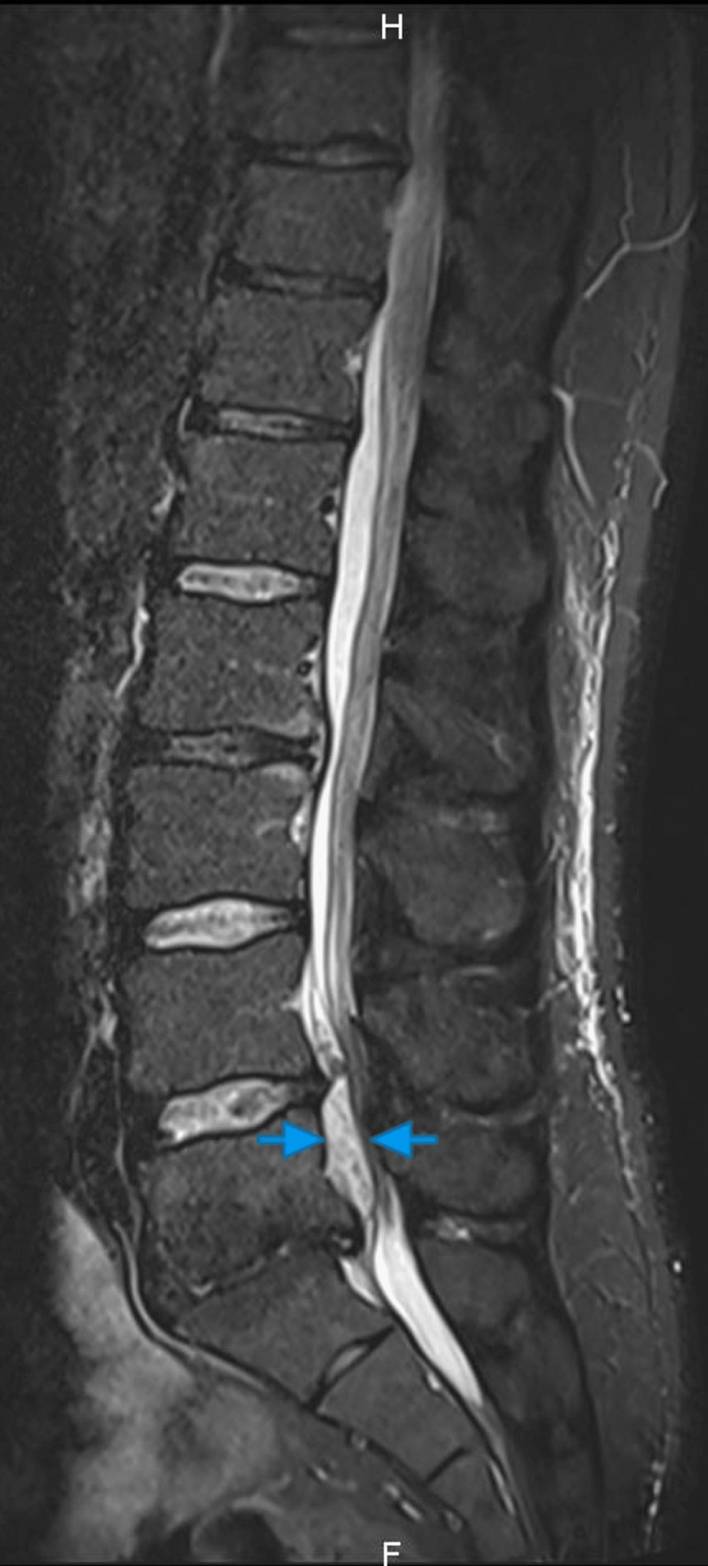
Sagittal T2-weighted fat-saturation image. Sagittal T2-weighted, fat-saturation image showing spinal cord compression by a mixed intensity structure at L4-L5 level. The structure is delineated by two blue arrows on each side.

**Figure 3:**
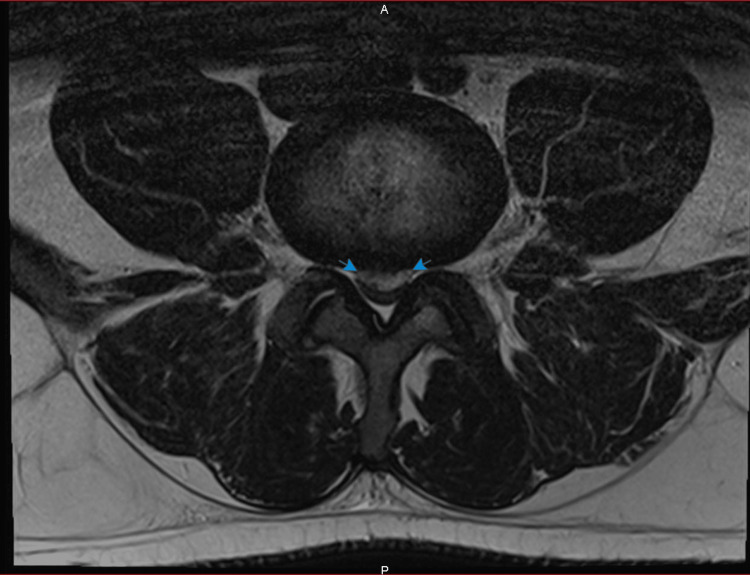
Axial T2-weighted image. Axial T2-weighted image showing the mass compressing the spinal cord at L4-L5 level. The structure is delineated by two blue arrows on each side.

The MRI was done when he presented again and showed a T1 iso-signal (Figure 1), T2 mixed hypo-/hyper-signal (Figure 2-3) longitudinal mass extending from L4 to S1, elevating the anterior epidural space and compressing the cauda equina roots, particularly the left L5 and S1 roots.

**Figure 4:**
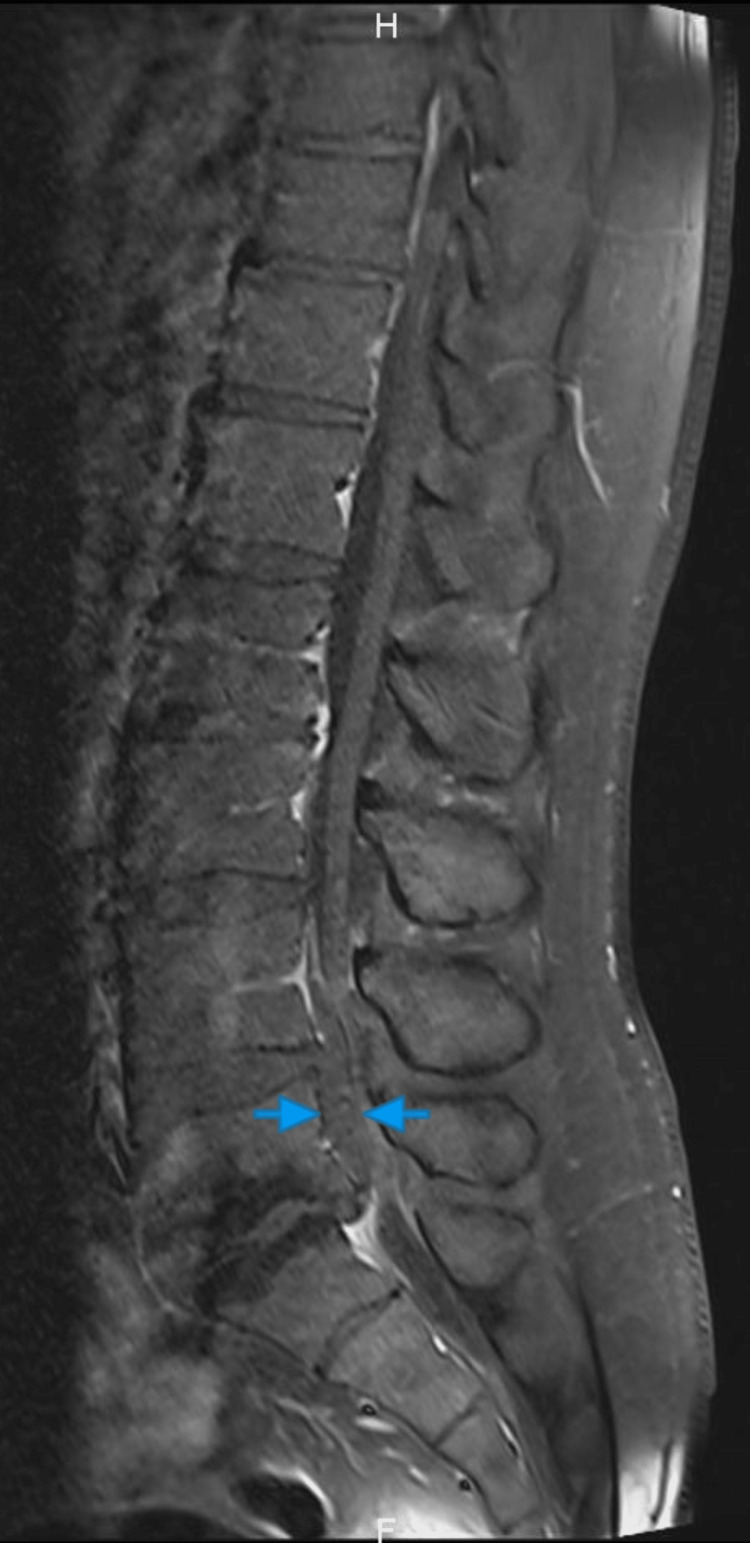
T1-weighted sagittal cut with gadolinium injection. No enhancement is observed in or around the mass, ruling out epiduritis. The mass is indicated by blue arrows.

After gadolinium injection, no enhancement was observed in the epidural region (Figure 4) excluding epiduritis. The radiology report did not provide a definitive distinction between a compressive hematoma or an extruded nucleus pulposus. It is worth noting that the mass present on the MRI presented the same signal intensity as the L4-L5 disc material, mainly NP.

**Figure 5:**
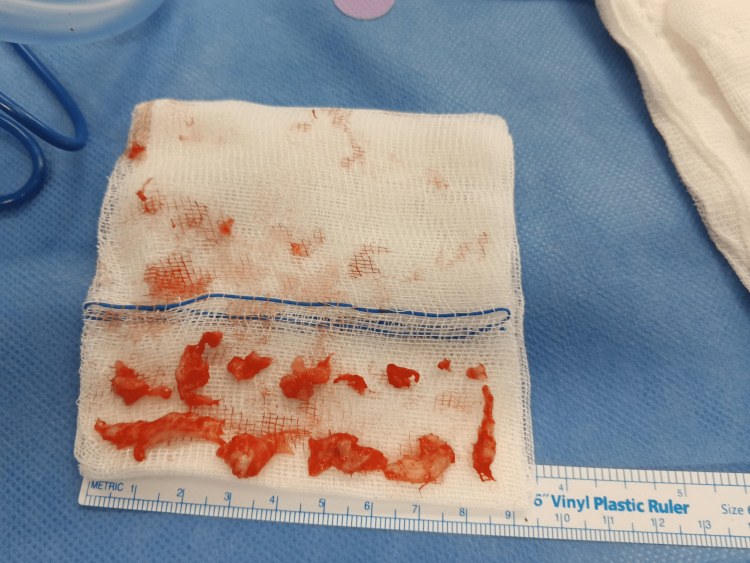
Fragments removed during the operation.

Six hours after presentation, surgical intervention was conducted. Utilizing a posterior approach, a 10 cm skin incision was made at the L4-L5 level, followed by a longitudinal opening of the aponeurosis. The gutters were then released up to the transverse processes while respecting the subperiosteal space on the left side. Subsequently, the ligamentum flavum on the left side between L4 and L5 was opened, along with a hemilaminectomy of L5. The dural sac and L5 root were reclined medially, revealing a significant extrusion of disc material from the L4-L5 disc space. This extrusion occupied the anterior left side of the spinal canal from L4 to S1, consistent with the findings in the MRI. Multiple fragments anterior to the spinal cord and causing compression were extracted (Figure 5). These fragments were confirmed to be extruded nucleus pulposus through histopathological examination. A complete L4/L5 herniotomy was performed via an L5 hemilaminectomy. The procedure was successfully completed, and 4 mg of Dexamethasone was locally administered to the right L5 nerve root.

After the operation, it was discovered that the patient had a neurogenic bladder. Sensory and motor symptoms remained similar, but the shooting and radiating pain had resolved.

Six months post-surgery, the patient's urinary symptoms improved, motor power was rated ⅘ in muscles involved in hip abduction and ankle dorsiflexion, the anal reflex was still negative, but the anal tone had improved, and sensory symptoms had disappeared.

## Discussion

A herniated disc is when the nucleus pulposus, or part of it, protrudes outside the annulus fibrosus and into the spinal canal. The symptoms can vary from no noticeable signs to radiculopathies, which involve motor, sensory, or mixed nerve root compression, and in extreme cases, cauda equina syndrome. This condition arises due to nerve compression and inflammation, unlike mechanical back pain, which lacks radiating pain, shooting sensations, and neuromuscular deficits [2].

The most common cause of a herniated disc is degenerative changes that lead to dehydration of the nucleus pulposus. The second most frequent cause is after an acute traumatic event. In most cases, conservative treatment is attempted, as herniations tend to heal within eight to 12 weeks. However, in refractory cases, severe or progressive symptoms require surgical intervention [7].

On the other hand, SEH is one of four types of spinal hematomas, alongside subdural, subarachnoid, and intramedullary hematomas. SEH refers to the collection of blood between the dura mater and the posterior wall of the vertebral body. The causes of SEH can be iatrogenic, spontaneous, or traumatic [8]. Spontaneous SEH can result from increased intraspinal pressure during activities like Valsalva maneuvers, weight lifting, or labor.

Symptoms of SEH can vary from being asymptomatic and resolving without detection to experiencing a sudden onset of back pain with progressive paresis or paresthesia, which can lead to partial or complete paralysis if left untreated, and death especially if it is located in the cervical spine [9]. Typically, SEH deteriorates gradually between 15 and 72 hours because of the venous origin of the bleeding in the majority of cases [10]. The location of SEH is often posterior or posterolateral in the spinal canal in 84% of cases and anterior in 12%.

MRI is the imaging technique of choice for assessing both disc herniations and SEH [11], as it provides precise information about the location, compression, and relationship to the surrounding structures of the affected area [12]. Signal intensity on MRI differs between disc herniations and hematomas and also varies depending on the chronicity of the lesion. SEH typically appears iso or hypointense on T1 within the first 24 hours and exhibits heterogeneity or mixed high and low signals on T2 [13] whereas disc herniations appear hyperintense on T1 and hypointense on T2 [14].

In our case, the MRI findings were a T1 hypointense, and T2 mixed hypo-hyper intense mass. This finding, with the progressive nature of the symptoms over more than 72 hours, led to our conclusion that this might be a SEH more likely than disc herniation. But we could not eliminate a disc herniation due to the possibility of signal change of the latter depending on the chronicity and the cause of the lesion [15] and the continuity with the disc space. Either way, symptoms of cauda equina led us to urgent operative treatment regardless of the possible etiology. During the operation, we found out that it is a massive disc extrusion without any signs of hematoma.

In the literature, we found five other cases with diagnostic ambiguity like ours. We added our case to the tables done by Basile et al. and Kim et al. [16, 17] (Table 1).

**Table 1:** An analysis of clinical and MRI findings in the literature related to disc herniation cases that is resembling an epidural hematoma. T1WI = T1-weighted image, T2WI = T2-weighted image, Mixed = mixed intensity low/high. *This information is not present in the articles nor described in the texts or legends. It was concluded from the images present in the articles.

Author/y	Age, gender	Trauma history	Level	T1WI	T2WI	Disc herniation pattern	Additional findings	Surgery	Connection with the disc space*
Nader et al. 2023 (present case)	38 yo, M	Axial trauma, deadlift	L4-L5	Hypo	Mixed	Upward and downward migration, longitudinal	No enhancement on injected MRI	Hemilaminectomy L5, microdiscectomy L4-L5	Yes. On all the images present in the different studies we found a communication with the disc space. But this information is not given in the articles, nor in the legends of the images
Basile et al. 2019 [16]	27 yo, M	High-energy fall spinal trauma	L4-L5	Mixed (medium/high)	High	Downward below L5 root, ventral to dura, upward migration	Longitudinal shape	Hemilaminectomy L4-L5, foraminotomy L4-L5 and L5-S1, microdiscectomy L4-L5	
Kim et al. 2019 [17]	39 yo, M	Crushing injury due to crane collapse	L3-L4	Iso (peripheral hyperintensity on T1WI)	Mixed		Longitudinal shape burst fracture of L4	Hemilaminectomy L3 and percutaneous screw fixation L3-L5	
Jain et al. 2018 [18]	50 yo, M	Hit his back on a tree branch	L2-L3	Iso (peripheral hyperintensity on T1WI)	Mixed		Longitudinal shape	Laminectomy L2-L3, fragment removal	
Song et al. 2012 [6]	23 yo, M	Traffic accident	L4-L5	Low (high signal rim around mass on T1WI)	Mixed	Ventral to dura		Bilateral hemilaminectomy L4, fragment removal	
Kil and Park 2017 [19]	57 yo, M	Epidural nerve block	L2-L3	Iso	Low	Posterior, dorsolateral to dura		Laminectomy L2, fragment removal	No, based on the fact that it is posterior to the dura, not anterior

## Conclusions

On magnetic resonance imaging, spinal epidural hematomas and acute lumbar disc herniations can cause diagnostic challenges. However, the key to differentiate between the two conditions lies in carefully correlating the clinical symptoms and signs with the imaging findings. When patients present with symptoms suggestive of neural compression, such as cauda equina syndrome or radiculopathy, and there is uncertainty regarding the precise etiology based on imaging alone, surgical intervention becomes essential to relieve neural compression and address the underlying cause.

Therefore, careful clinical evaluation and the consideration of surgical intervention based on the patient's presentation are essential steps in effectively managing cases where SEH is suspected. Timely surgical intervention can be vital in providing accurate diagnosis, alleviating neural compression, and promoting a favorable outcome for the patient.

## References

[1] Al Qaraghli MI, De Jesus O (2023). Lumbar disc herniation. https://www.ncbi.nlm.nih.gov/books/NBK560878/.

[2] Dydyk AM, Ngnitewe Massa R, Mesfin FB (2023). Disc herniation. https://www.ncbi.nlm.nih.gov/books/NBK441822/.

[3] Kasra M, Shirazi-Adl A, Drouin G (1992). Dynamics of human lumbar intervertebral joints. Experimental and finite-element investigations. Spine (Phila Pa 1976).

[4] Al-Mutair A, Bednar DA (2010). Spinal epidural hematoma. J Am Acad Orthop Surg.

[5] Wassenaar M, van Rijn RM, van Tulder MW (2012). Magnetic resonance imaging for diagnosing lumbar spinal pathology in adult patients with low back pain or sciatica: a diagnostic systematic review. Eur Spine J.

[6] Song K-J, Lee K-B, Kim D-Y (2012). A traumatic disc herniation mimicking an epidural hematoma in a young adult - a case report. Neurosurg Q.

[7] Carlson BB, Albert TJ (2019). Lumbar disc herniation: what has the Spine Patient Outcomes Research Trial taught us?. Int Orthop.

[8] Mohamed EH, Dsouza LB, Elnabawy WA, Bashir K, Elmoheen A (2020). Acute spinal extradural hematoma and cord compression: case report and a literature review. Cureus.

[9] Domenicucci M, Mancarella C, Santoro G, Dugoni DE, Ramieri A, Arezzo MF, Missori P (2017). Spinal epidural hematomas: personal experience and literature review of more than 1000 cases. J Neurosurg Spine.

[10] Unnithan AKA (2019). A brief review of literature of spontaneous spinal epidural hematoma in the context of an idiopathic spinal epidural hematoma. Egypt J Neurosurg.

[11] Johnson SM, Shah LM (2019). Imaging of acute low back pain. Radiol Clin North Am.

[12] Lavi ES, Pal A, Bleicher D, Kang K, Sidani C (2018). MR imaging of the spine: urgent and emergent indications. Semin Ultrasound CT MR.

[13] Moriarty HK, O Cearbhaill R, Moriarty PD, Stanley E, Lawler LP, Kavanagh EC (2019). MR imaging of spinal haematoma: a pictorial review. Br J Radiol.

[14] Konieczny MR, Reinhardt J, Prost M, Schleich C, Krauspe R (2020). Signal intensity of lumbar disc herniations: correlation with age of herniation for extrusion, protrusion, and sequestration. Int J Spine Surg.

[15] Tarukado K, Ikuta K, Fukutoku Y, Tono O, Doi T (2015). Spontaneous regression of posterior epidural migrated lumbar disc fragments: case series. Spine J.

[16] Basile L, Brunasso L, Gerardi RM (2020). Traumatic lumbar disc extrusion mimicking spinal epidural hematoma: case report and literature review. Surg Neurol Int.

[17] Kim JH, Kim SH, Lee SK, Moon BJ, Lee JK (2019). Traumatic lumbar disc herniation mimicking epidural hematoma: a case report and literature review. Medicine (Baltimore).

[18] Jain N, Crouser N, Yu E (2018). Lumbar intervertebral disc herniation masquerading as an epidural hematoma: a case report and review of the literature. JBJS Case Connect.

[19] Kil JS, Park JT (2017). Posterior epidural herniation of a lumbar disk fragment at L2-3 that mimicked an epidural hematoma. Korean J Spine.

